# Supplementary figures and images for: Inequalities in Immunization against Maternal and Neonatal Tetanus: A Cross-Sectional Analysis of Protection at Birth Coverage Using Household Health Survey Data from 76 Countries
Source: Vaccines (Basel). 2023 Mar 29;11(4):752. doi: 10.3390/vaccines11040752 (PMC10146835; doi:10.3390/vaccines11040752)

Countries that have achieved MNTE

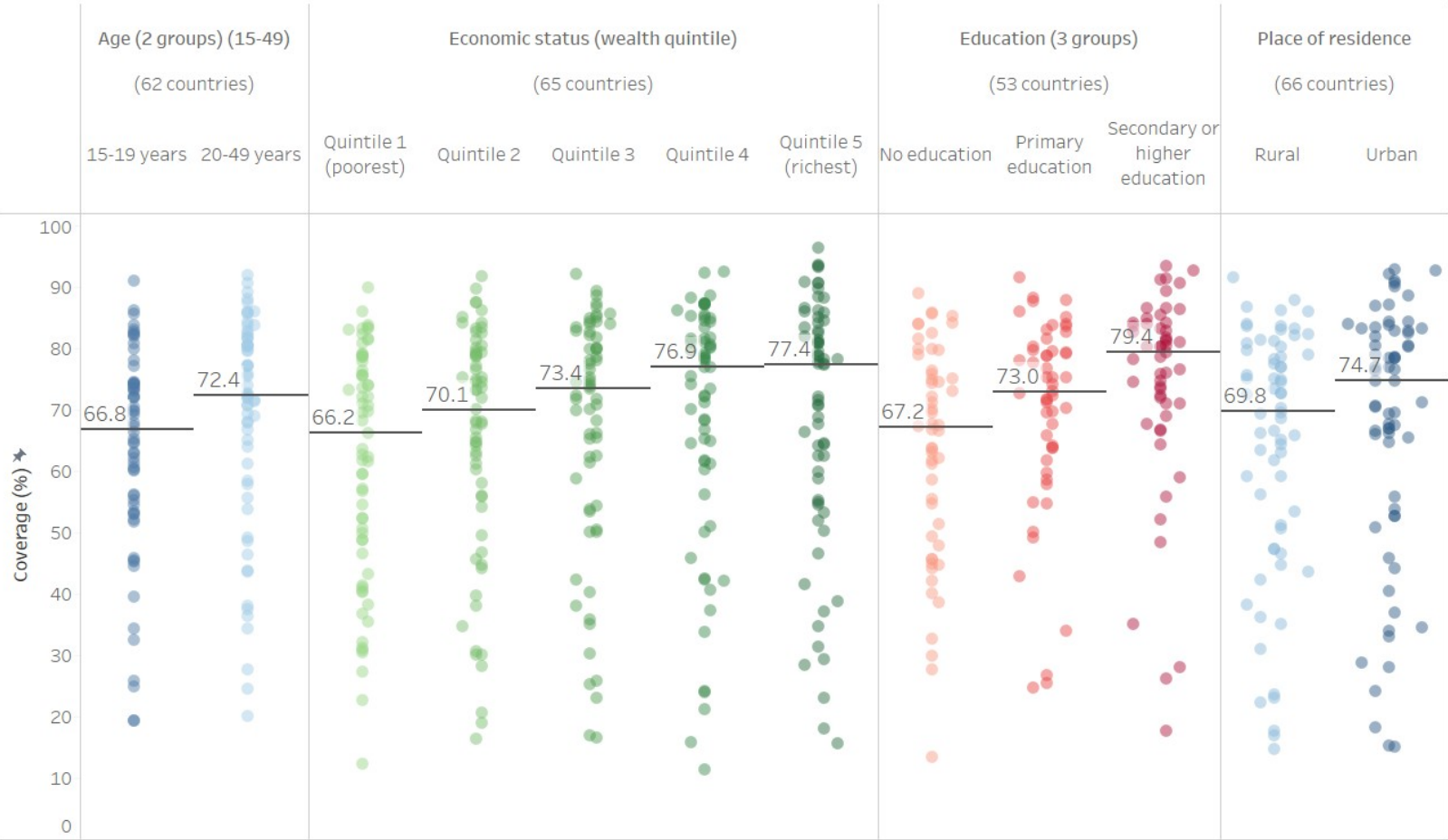

Countries that have not achieved MNTE

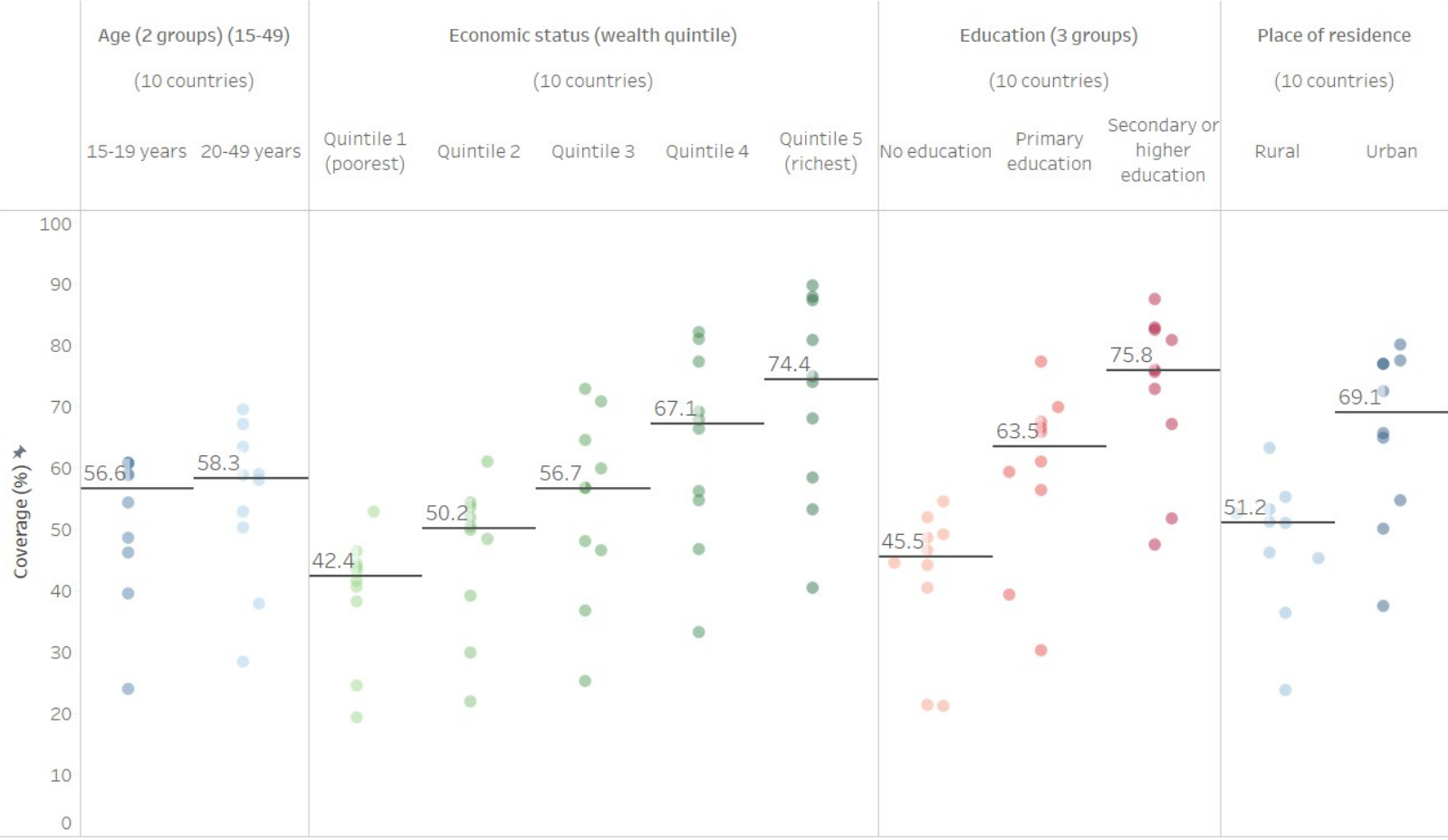

Supplement: Supplementary file 1 [file vaccines-11-00752-s001.zip › Figure S1.pdf]
